# Virtual Reality Reduces Pediatric Anxiety During Food Allergy Clinical Trials: A Pilot Randomized, Pragmatic Study

**DOI:** 10.3389/falgy.2021.779804

**Published:** 2022-01-13

**Authors:** Sarah Alonzi, Thomas J. Caruso, Sayantani B. Sindher, Shu Cao, Sara Varadharajulu, William J. Collins, R. Sharon Chinthrajah

**Affiliations:** ^1^Department of Psychology, University of California, Los Angeles, Los Angeles, CA, United States; ^2^Department of Anesthesiology, Perioperative, and Pain Medicine, Stanford University School of Medicine, Stanford, CA, United States; ^3^Sean N. Parker Center for Allergy and Asthma Research at Stanford University, Stanford University, Stanford, CA, United States; ^4^Department of Medicine, University of California San Francisco School of Medicine, San Francisco, CA, United States; ^5^Department of Medicine, Division of Hospital Medicine, Stanford University School of Medicine, Stanford, CA, United States; ^6^Division of Pulmonary and Critical Care Medicine, Stanford University, Stanford, CA, United States

**Keywords:** food allergy, virtual reality, phlebotomy, anxiety, fear, pain

## Abstract

Phlebotomy procedures required in food allergy (FA) diagnosis and clinical trials often induce fear and anxiety for pediatric patients. The primary aim of this study was to determine whether virtual reality (VR) applications were effective in reducing anxiety for pediatric FA patients undergoing phlebotomy during FA clinical trials. Secondary aims assessed fear, pain, procedural compliance, and adverse events. Participants undergoing phlebotomy were enrolled and randomized to a VR group or standard of care (SOC) group for this prospective pilot randomized, pragmatic study. Participants in the VR group played interactive applications on a customized Samsung Gear VR headset and those in the SOC group received the standard of care. Participants' anxiety, fear, and pain were assessed with the Children's Anxiety Meter, Children's Fear Scale, and FACES pain scale pre, during, and post phlebotomy procedure. Compliance was assessed using the modified Induction Compliance Checklist during the procedure and compared between two groups. Forty-nine participants were randomized to VR (*n* = 26) and SOC (*n* = 23) groups. Although both the VR and SOC groups experienced a decrease in anxiety and fear from pre- to post-procedure, those in the VR group experienced less anxiety and fear during the procedure than SOC participants. Similarly, both groups experienced an increase in pain from pre- to post-procedure; however, the VR group reported less pain during the procedure than SOC. Fewer symptoms of procedural non-compliance were reported in the VR group. Interactive VR applications may be an effective tool for reducing fear, anxiety, and pain during phlebotomy for FA clinical trials.

## Introduction

Food allergies (FA) are increasingly prevalent globally (1), affecting up to 6% of children in the United States (2, 3). While oral food challenge (OFC) is the “gold standard” for food allergy diagnoses, comprehensive diagnostic testing often requires skin prick testing or serum-specific immunoglobulin E (IgE) (4, 5). Serum IgE testing requires phlebotomy, which often invokes fear and anxiety in children (6, 7). In addition to diagnostic testing, frequent phlebotomy is requisite for many pediatric allergy clinical trials.

Needle phobia is more common among children than adults, with fifty to sixty percent of children reporting fear of needles (8–10). Needle phobia is associated with treatment avoidance, non-adherence with vaccine schedules, and anticipatory unpleasant side effects (6, 11, 12). Previous negative experiences with phlebotomy during childhood may result in medical treatment aversion into adulthood (13). FA patients are particularly at risk for repercussions from needle phobia as most anaphylaxis deaths are caused by delayed administration of epinephrine due to fear of injection (14, 15). Additionally, needle phobia may contribute to reluctance toward routine diagnostic testing and participation in FA trials that require phlebotomy.

Virtual reality (VR) has been used to reduce pediatric anxiety and pain for patients with burns and those undergoing minor procedures (16–18). Although this immersive technology has been leveraged to improve physical and emotional distress in a variety of medical settings (19), there is limited research of VR in FA patients. Given the prevalence of pediatric needle phobia and increasing incidence of pediatric FA, the goal of this study was to determine if VR was a useful tool to improve the experience of children with FA. The primary aim was to determine if VR reduced anxiety. The secondary aims examined fear, pain, procedural compliance, and adverse events.

## Methods

### Study Design

In this prospective, pilot randomized study, FA patients undergoing phlebotomy at an academic allergy research center in Northern California were randomly assigned to a VR or standard of care (SOC) group using a random number generator in REDCap, with 1:1 allocation (20). This study was approved by the Stanford University Internal Review Board and registered prior to the enrollment of the first patient (NCT 03628989). Inclusion criteria were patients 6–17 years old undergoing FA-related phlebotomy. Patients were excluded if they did not speak English or had a history of seizure, motion sickness, or severe developmental delay.

### Intervention

Prior to enrollment, eligible patients undergoing phlebotomy were screened by a trained research assistant for exclusion criteria. Following written parental informed consent and patient assent, participants were enrolled, completed a baseline questionnaire, and then randomized to VR or SOC. In the VR group, participants received topical numbing medicine and played interactive applications on a customized, infection control compliant Samsung Gear VR headset (Samsung, Seoul, South Korea) in conjunction with a Samsung S7 or S8 mobile device during phlebotomy. VR participants received an explanation of how to use the headset and began the application ~1 min before vascular access. For SOC participants, they received topical numbing medicine, parental distraction, and toy play per patient preference. Although patients were attending the allergy research center for varying FA procedures (e.g., allergy testing, oral food challenge, oral immunotherapy, etc.), phlebotomy was completed prior to any visit-specific procedures, and research assistants were trained to inquire about participants' phlebotomy-related anxiety, fear, and pain only, to limit the influence of other visit-related distress on responses.

### Outcomes

The primary outcome was to determine whether anxiety scores differed between groups. Anxiety was measured using the Children's Anxiety Meter (CAM) (21) at baseline, during the procedure, and ~1 min after the procedure. The CAM is a self-reported, vertical analog measure of anxiety with values ranging from zero “calm: not nervous or worried” to ten “very, very nervous or worried (21).”

The secondary outcomes of fear, pain, and procedural compliance were measured using the Children's Fear Scale (CFS), pain FACES scale, and modified Induction Compliance Checklist (mICC), respectively. CFS is a validated, self-reported measure of fear with values ranging from 0 to 4 and corresponding faces depicting the level of fear (22). FACES is a pain rating scale ranging from 0 to 10 with corresponding facial expression graphics (23). mICC is based on a 10-item observer-rated checklist of behaviors that interfere with anesthesia (e.g., screaming, moving away from clinicians, and requiring physical restraint), modified for phlebotomy, with higher scores indicating poorer compliance (24). The CFS and FACES ratings were self-reported and measured at three intervals: baseline, during procedure, and ~1 min after the procedure. A trained research assistant completed the mICC during the procedure. Incidence of adverse events were monitored throughout the study.

### Statistical Analysis

Descriptive statistics including mean and standard deviation (SD) for continuous variables and frequencies and proportions for categorical variables were used to describe cohort demographics, endpoints, and incidence of adverse events. Two-sample *t*-test was also applied to compare the baseline anxiety, fear, and pain between two groups. The change in anxiety, fear, and pain from baseline to during and post-procedure were assessed using the linear mixed-effect model as a function of time points, treatment, time and treatment interaction, age in years, and with a participant level random effect. The change of endpoints was also explored within each group. Procedural study data were collected and stored using REDCap electronic data capture tools hosted at Stanford University. Analyses were performed using R version 3.6.0.

## Results

### Recruitment and Participant Flow

A total of 63 patients qualified for study eligibility and were recruited from June 18, 2019 to August 16, 2019 (Supplementary Figure 1). Forty-nine participants were randomized to a VR (*n* = 26) or SOC (*n* = 23) group. Two participants randomized to VR were excluded due to non-compliance. Patients were 44.7% female, with a mean age of 11 years, and the majority (95.5%) reported previously undergoing a similar procedure (Supplementary Table 1).

### Outcomes

**Figure 1:**
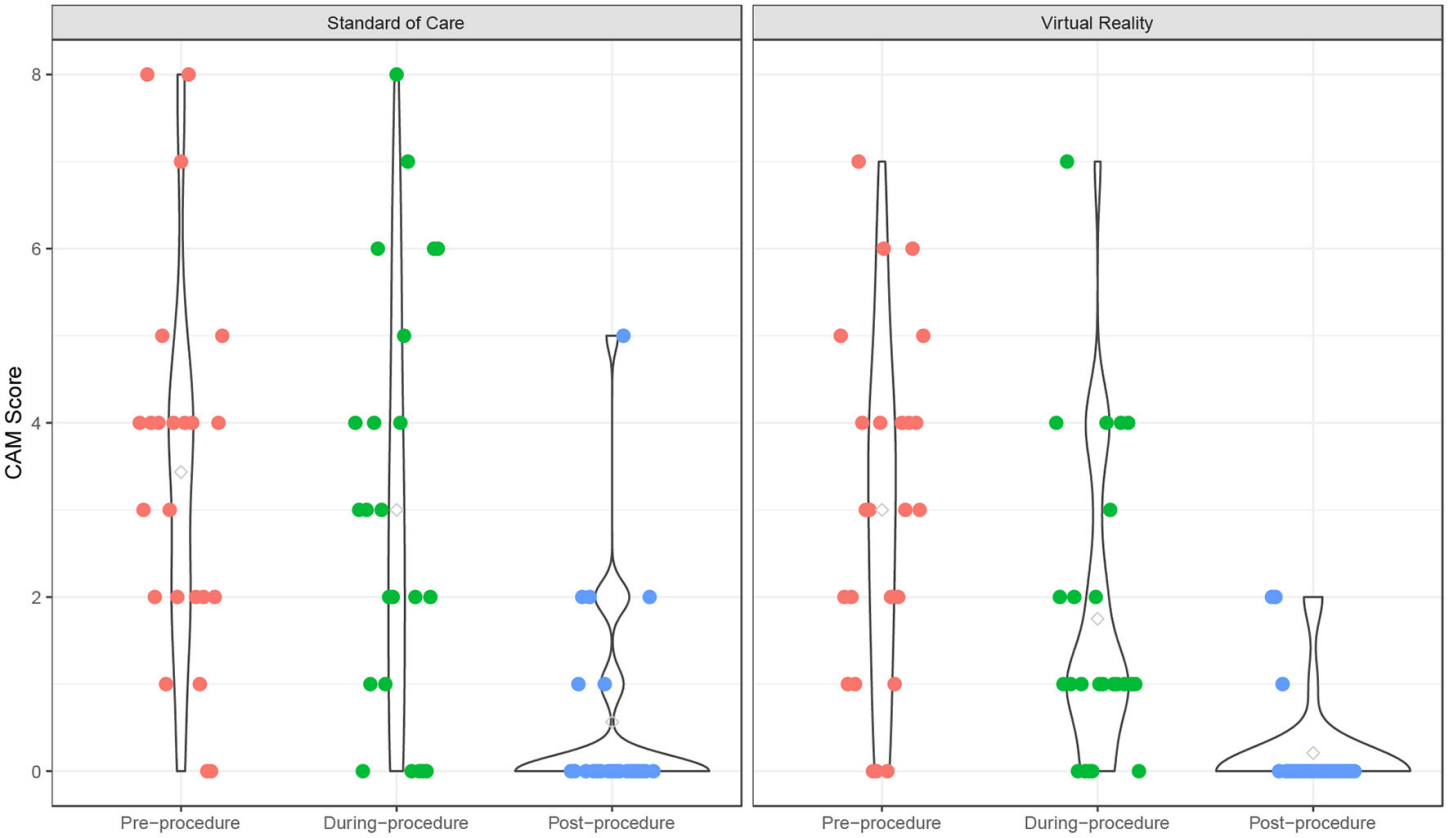
Children's Anxiety Meter over time. Anxiety scores of Virtual Reality (VR) and SOC groups as measured by the Children's Anxiety Meter (CAM).

Before the procedure, the anxiety, fear, and pain levels were comparable between two groups. Participants experienced a significant decrease of anxiety from pre- to post-procedure {time point effect coefficients [95% confidence interval (CI)]: from pre- to during-procedure: −0.43 (−1.32 to −0.45); pre- to post-procedure: −2.87 (−3.75 to −1.99); *p* < 0.001}, and this trend was similar in both VR (*p* < 0.001) and SOC (*p* < 0.001) groups (Supplementary Table 2; Figure 1). The time effect on anxiety was comparable between the two groups (interaction term *p* = 0.35). There was no significant age effect on anxiety levels (*p* = 0.07). During the procedure, VR participants experienced less anxiety [mean (M) = 1.75, standard deviation (SD) = 1.75] than SOC (*M* = 3.00, SD = 2.45; *p* = 0.05). From baseline to post-procedure measure, VR participants experienced a 41.7% mean decrease in anxiety (*M* = −1.25, SD = 2.49, *p* = 0.024), compared to a 12.5% decrease for SOC (*M* = −0.43, SD = 2.21, *p* = 0.36).

**Figure 2:**
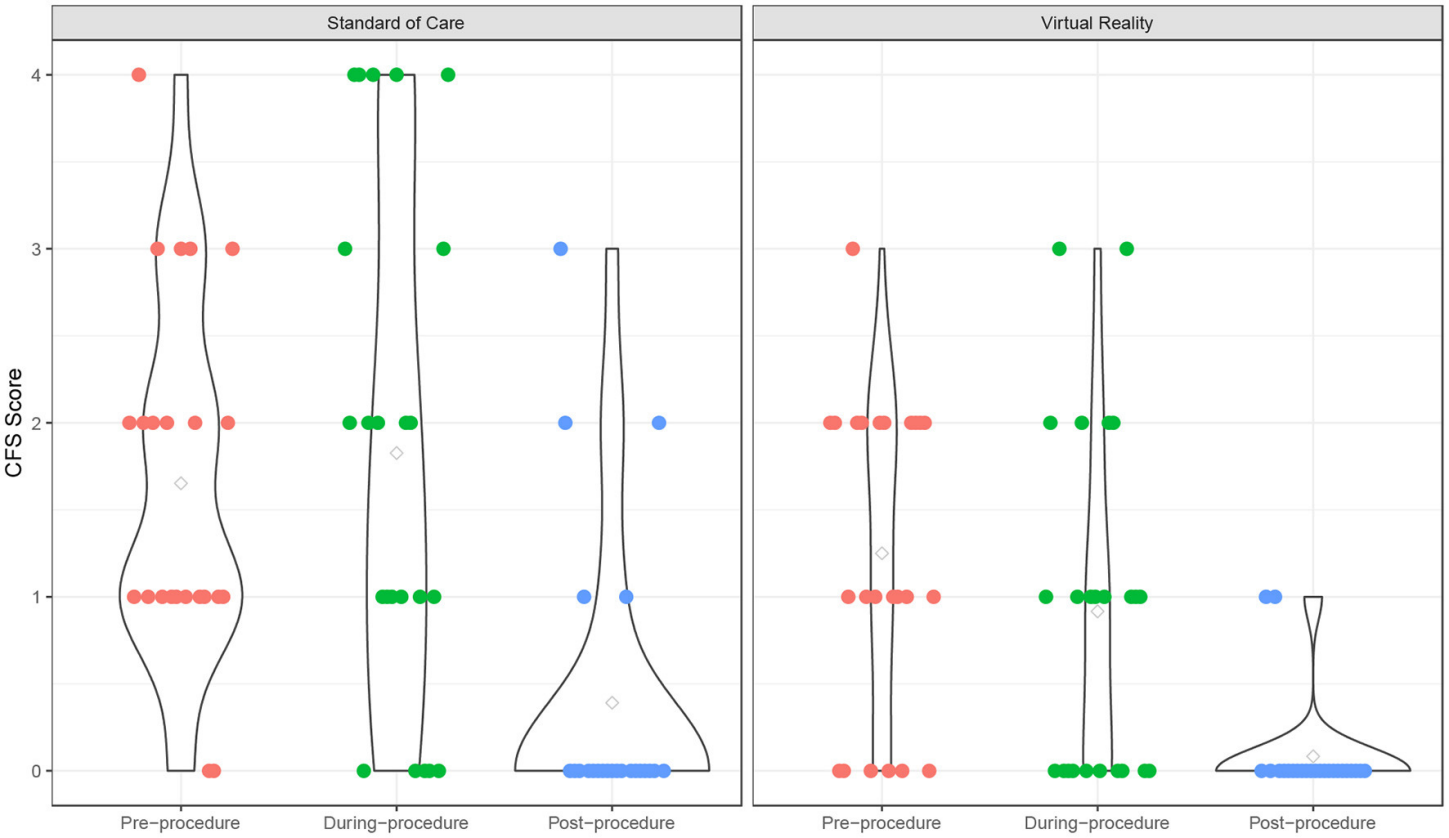
Fear score over time. Fear scores of Virtual Reality (VR) and SOC groups as measured by the Children's Fear Scale (CFS).

**Figure 3:**
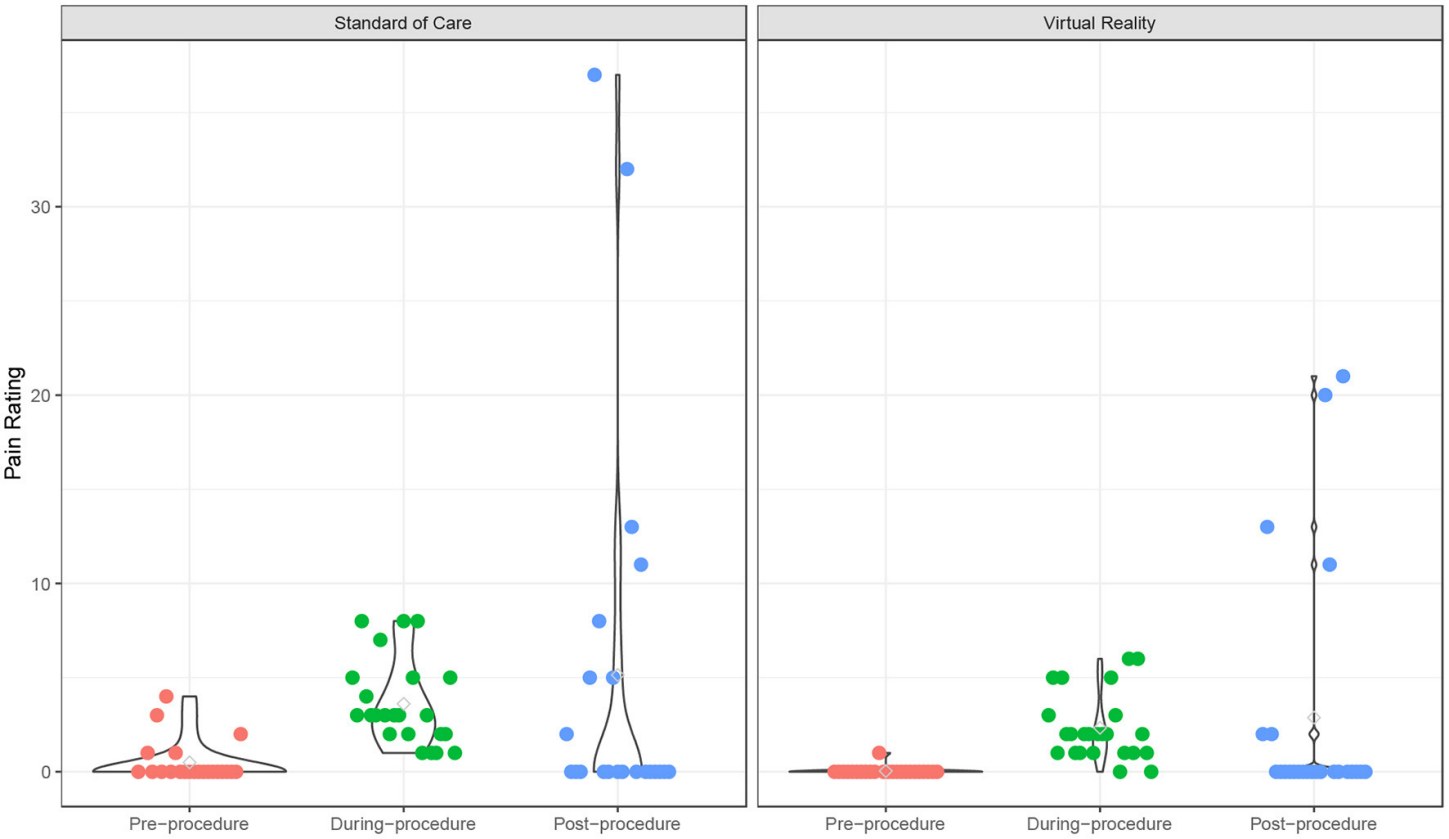
Pain rating over time. Pain ratings of Virtual Reality (VR) and SOC groups as measured by the Pain scale.

**Figure 4:**
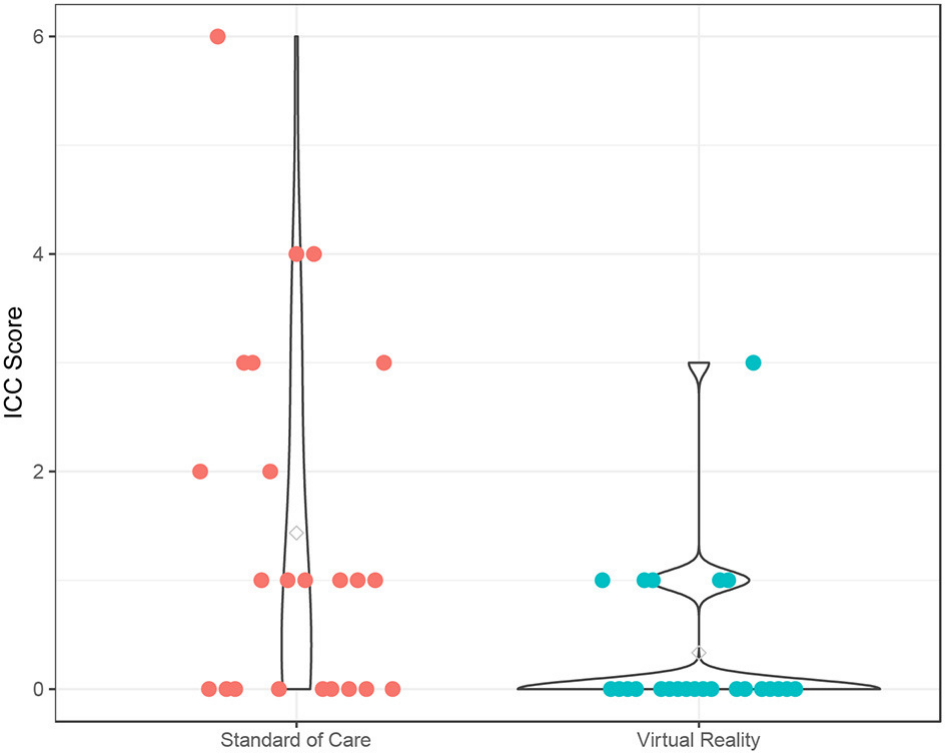
Procedural Compliance. Procedural Compliance of Virtual Reality (VR) and SOC groups as measured by the modified Induction Compliance Checklist (mICC). Higher scores on the ICC indicate less procedural compliance.

The fear scores also declined from pre- to post-procedure (*p* < 0.001), and the VR group experienced a significantly lower fear score over time compared to the SOC group (*p* = 0.049) (Supplementary Table 2; Figure 2). The time effect on fear score was comparable between the two groups (interaction term *p* = 0.18). There was no significant age effect on the fear score (*p* = 0.18). In addition, participants experienced a significant increase in pain from pre- to post-procedure (*p* < 0.001), and this trend was similar in both VR (*p* = 0.018) and SOC (*p* = 0.026) groups (Supplementary Table 2; Figure 3). The time effect on pain scores was comparable between the two groups (interaction term *p* = 0.68). There was no significant age effect on pain scores (*p* = 0.16). VR participants reported less pain (*M* = 2.33, SD = 1.79) than SOC (*M* = 3.61, SD = 2.29, *p* = 0.040) during the procedure. Additionally, lower mICC scores were reported among VR participants (*M* = 0.33, SD = 0.70) than SOC participants (*M* = 1.44, SD = 1.67, *p* = 0.007; Figure 4), indicating that VR participants experienced fewer symptoms of procedural non-compliance.

One patient in the VR group reported nausea. No other adverse events were reported.

## Discussion

In a sample of pediatric patients undergoing phlebotomy for FA testing, immersive VR applications were an effective tool for reducing fear, anxiety, and pain during phlebotomy. VR participants showed a greater decrease in anxiety and fear from baseline to post-procedure than SOC group. Patients who used VR experienced less pain and were significantly more compliant during phlebotomy.

The between-groups differences in reported anxiety, fear, and procedural compliance are consistent with other RCTs using VR in pediatric cohorts (16, 17). Our finding that VR patients experienced less pain during phlebotomy is consistent with studies of patients ranging from adolescents to young adults undergoing burn treatment and phlebotomy (16, 18). However, these results are in contrast to a larger study of pediatric patients undergoing vascular access, which did not find a significant difference in pain between VR and SOC groups (16–18).

This study has some limitations. First, all participants underwent phlebotomy at the same academic medical center with a small team of providers, which limits generalizability. The population was relatively homogenous, consisting of predominately Caucasian and Asian participants. Second—the SOC group included a variety of different anxiolytics, which limited control. However, as a pragmatic study, this may increase generalizability to other settings. Third, consistent with other VR studies, participants were instructed to choose between multiple applications, providing some measure of patient autonomy. Finally, we observed a wide standard deviation on our measures of anxiety, fear, and pain. Although this could be due to the small sample size, it is noteworthy that the wide dispersion of scores could indicate that VR is a more effective distress-reducing technology among certain patient groups. As such, the study provides justification for the exploration of patient-level factors that might moderate the effects of VR on anxiety, pain, fear, and procedural compliance in larger RCTs that are sufficiently powered to detect these effects. For example, future research with larger sample sizes could examine intervention effectiveness between patients with high and low socioeconomic status, those exposed to VR games with and without music, and perform a more rigorous analysis of intervention effects by patient age groups.

VR demonstrated the potential to increase patient satisfaction during routine allergy testing, which may also increase patient retention in clinical trials studies which are imperative to FA diagnoses and treatments. Future studies will leverage the effect size demonstrated in this pilot to design larger powered studies and include broader applications during additional allergy procedures, such as skin prick testing and food challenges.

## Data Availability Statement

The original contributions presented in the study are included in the article/Supplementary Files, further inquiries can be directed to the corresponding author/s.

## Ethics Statement

The studies involving human participants were reviewed and approved by Stanford University Internal Review Board. Written informed consent to participate in this study was provided by the participants' legal guardian/next of kin.

## Author Contributions

SA contributed to data collection, preliminary analyses, and manuscript writing. TC developed the study protocol and contributed to the manuscript writing and editing. SS supervised the data collection and contributed to manuscript writing. SC verified the statistical methods, contributed to statistical and methodological writing, and designed the final manuscript figures. SV contributed to data collection and data analysis. WC supervised data collection and contributed to manuscript writing and editing. RC supervised the data collection, regulatory affairs, and manuscript writing. All authors contributed to the article and approved the submitted version.

## Funding

This work was supported by the National Institutes of Health (U19AI104209), the Sean N. Parker Center for Allergy and Asthma Research at Stanford University, the Department of Pediatrics at Stanford Medicine, Lucile Packard Children's Hospital, Child Health Research Institute (CHRI), Food Allergy Research and Education (FARE) Center of Excellence, Myra Reinhard Foundation, End Allergies Together (EAT), the Hartman Family Foundation, and the Naddisy Foundation.

## Conflict of Interest

The authors declare that the research was conducted in the absence of any commercial or financial relationships that could be construed as a potential conflict of interest.

## Publisher's Note

All claims expressed in this article are solely those of the authors and do not necessarily represent those of their affiliated organizations, or those of the publisher, the editors and the reviewers. Any product that may be evaluated in this article, or claim that may be made by its manufacturer, is not guaranteed or endorsed by the publisher.

## Abbreviations

Abbreviations: FA: Food Allergy
M: Mean
OFC: Oral Food Challenge
SD: Standard Deviation
SOC: Standard of Care
VR: Virtual Reality.
