# Supplementary material for: Virtual Reality Reduces Pediatric Anxiety During Food Allergy Clinical Trials: A Pilot Randomized, Pragmatic Study
Source: Front Allergy. 2022 Jan 13;2:779804. doi: 10.3389/falgy.2021.779804 (PMC8974765; doi:10.3389/falgy.2021.779804)
Supplement: Supplementary file 1 [file Table_1.docx]

Supplementary Material

# Supplementary Figures and Tables

## Supplementary Figures


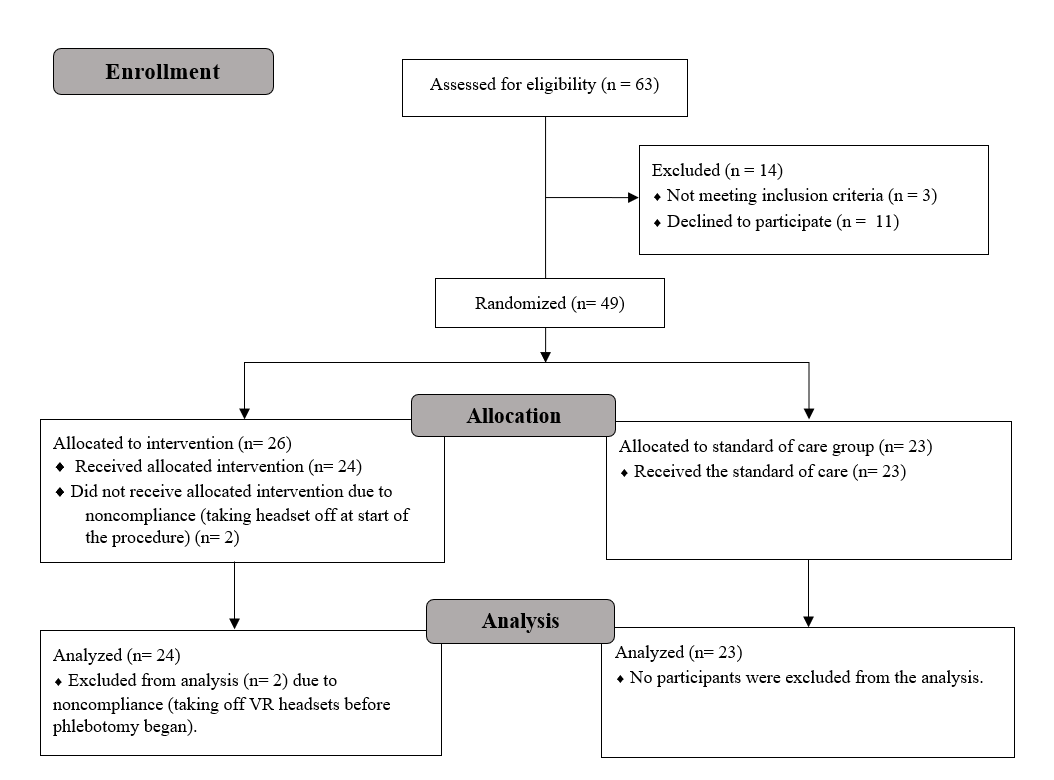


**Supplementary Figure 1.** Participant Flow Diagram to illustrate the prosses by which participants were recruited, allocated to study groups, and which data were analyzed.

**1.2 Supplementary Tables**

**Supplementary Table 1: Participant Characteristics**

|  |  | **Arm** | |
| --- | --- | --- | --- |
|  |  | **Standard of Care** | **Virtual Reality*** |
| **N** | **47** | **23** | **24** |
| Female (%) | 21 (44.7%) | 13 (56.5%) | 8 (33.3%) |
| Age | 11.33 (2.88) | 10.39 (2.33) | 12.26 (3.12) |
| Non-Hispanic (%) | 45 (95.7%) | 23 (100%) | 22 (91.7%) |
| **Race** (%) |  |  |  |
| Caucasian | 20 (42.6%) | 12 (52.2%) | 8 (33.3%) |
| Black | 1 (2.1%) | 1 (4.3%) | 0 |
| Asian | 19 (40.4%) | 9 (39.1%) | 10 (41.7%) |
| Native Hawaiian or Pacific Islander | 1 (2.1%) | 1 (4.3%) | 0 |
| Prefer not to report | 6 (12.8%) | 5 (21.7%) | 1 (4.2%) |
| **Similar Experiences** (%) |  |  |  |
| Had previous similar procedure | 42 (95.5%) | 19 (95.0%) | 23 (95.8%) |
| Used VR before | - | - | 17 (70.8%) |

Mean and standard deviation are presented for continuous variables; percentages are presented for categorical variables.

* Two patients were randomized and excluded from the VR group for taking off headsets before the procedure.

**Supplementary Table 2: Clinical Outcomes.**

|  |  | **Arm** | |  |
| --- | --- | --- | --- | --- |
|  |  | **Standard of Care** | **Virtual Reality** |  |
| **N** | **47** | **23** | **24** |  |
| Pre procedure |  |  |  |  |
| Anxiety | 3.21 (2.08) | 3.43 (2.21) | 3.00 (1.98) |  |
| Fear | 1.45 (0.97) | 1.65 (1.03) | 1.25 (0.90) |  |
| Pain | 0.26 (0.79) | 0.48 (1.08) | 0.04 (0.20) |  |
| During procedure |  |  |  |  |
| Anxiety | 2.36 (2.19) | 3.00 (2.45) | 1.75 (1.75) |  |
| Fear | 1.36 (1.31) | 1.83 (1.47) | 0.92 (0.97) |  |
| Pain | 2.96 (2.13) | 3.61 (2.29) | 2.33 (1.79) |  |
| Post procedure |  |  |  |  |
| Anxiety | 0.38 (0.95) | 0.57 (1.20) | 0.21 (0.59) |  |
| Fear | 0.23 (0.63) | 0.39 (0.84) | 0.08 (0.28) |  |
| Pain | 3.96 (8.46) | 5.14 (10.28) | 2.88 (6.40) |  |
| Change from pre- to post procedure |  |  |  |  |
| Anxiety | -2.83 (2.15) | -2.87 (2.30) | -2.79 (2.04) |  |
| Fear | -1.21 (1.12) | -1.26 (1.29) | -1.17 (0.96) |  |
| Pain | 2.70 (2.13) | 4.77 (9.82) | 2.83 (6.42) |  |
| Change from pre- to during procedure |  |  |  |  |
| Anxiety | -0.85 (2.37) | -0.43 (2.21) | -1.25 (2.49) |  |
| Fear | -0.09 (1.10) | 0.17 (0.83) | -0.33 (1.27) |  |
| Pain | 2.70 (2.13) | 3.13 (2.36) | 2.29 (1.83) |  |

*Mean and standard deviation are presented for continuous variables.

* Two patients were randomized and excluded from the VR group for taking off headsets before the procedure.
